# Use of Mass-Participation Outdoor Events to Assess Human Exposure to Tickborne Pathogens

**DOI:** 10.3201/eid2303.161397

**Published:** 2017-03

**Authors:** Jessica L. Hall, Kathrin Alpers, Kevin J. Bown, Stephen J. Martin, Richard J. Birtles

**Affiliations:** University of Salford, Salford, UK

**Keywords:** *Borrelia*, Lyme borreliosis, *B. miyamotoi*, crowd sourcing, ticks, epidemiologic studies, public health, tickborne pathogens, vector-borne infections, Ixodes ricinus, bacteria

## Abstract

Mapping the public health threat of tickborne pathogens requires quantification of not only the density of infected host-seeking ticks but also the rate of human exposure to these ticks. To efficiently sample a high number of persons in a short time, we used a mass-participation outdoor event. In June 2014, we sampled ≈500 persons competing in a 2-day mountain marathon run across predominantly tick-infested habitat in Scotland. From the number of tick bites recorded and prevalence of tick infection with *Borrelia burgdoferi* sensu lato and *B. miyamotoi*, we quantified the frequency of competitor exposure to the pathogens. Mass-participation outdoor events have the potential to serve as excellent windows for epidemiologic study of tickborne pathogens; their concerted use should improve spatial and temporal mapping of human exposure to infected ticks.

The countryside (outdoors) represents a contemporary arena for recreation, and the benefits of such recreation to human health and well-being are widely recognized and strongly promoted by governments and other stakeholders. However, the countryside also harbors particular hazards that might be managed to minimize the threat they pose to countryside users. Among these hazards are numerous infectious diseases (e.g., Lyme borreliosis and leptospirosis) that are usually more abundant in the countryside than in urban areas. The public health burden of such infections can be defined as the product of the abundance of potential sources of infection (i.e., environmental hazards) and the frequency of human exposure (*1*). Although measuring both of these parameters presents difficulties, accurately quantifying the frequency of human exposure is particularly challenging.

Lyme borreliosis, caused by the spirochaete *Borrelia burgdorferi* sensu lato (s.l.), is a tickborne disease encountered primarily in the temperate regions of the Northern Hemisphere; in some countries, many thousands of cases are reported each year. For example, in the United States, ≈300,000 cases per year are estimated (*2*), and in the Netherlands, ≈25,000 new cases are reported each year (*3*). In the United Kingdom, Lyme borreliosis is less frequently reported; in 2014, the most recent annual data released by Public Health England (https://www.gov.uk/government/publications/lyme-borreliosis-epidemiology) and Health Protection Scotland (http://www.hps.scot.nhs.uk/giz/wrdetail.aspx?id = 65883&subjectid = 100&wrtype = 6) indicate almost 1,500 cases. In keeping with elsewhere in Europe, the incidence of cases in the United Kingdom has increased dramatically since the turn of the century and even now is considered a significant underestimation (*4*). The pathogenic potential of *Borrelia miyamotoi* was first reported in Russia in 2011; infections were most frequently manifested as an influenza-like illness (*5*). However, although the presence of this pathogen in *Ixodes* ticks across the Northern Hemisphere has been widely reported, reported cases of human disease are still rare (*6*) and have yet to be encountered in the United Kingdom. When estimating the public health burden of tickborne pathogens, the environmental hazard is represented by the abundance of infected ticks. Methods such as dragging and flagging for quantifying the abundance of questing *I. ricinus* nymphs and adults are well established and, although not without their shortcomings (*1*), have been widely adopted. However, methods quantifying human exposure to infected ticks are much more challenging. In the Netherlands, general practitioner consultations for tick bites have been used to monitor regional/national change in the frequency of tick bites (and thus tick activity) (*7*), but as most persons probably remove ticks themselves rather than rely on a general practitioner, this approach is a poor indicator of, at best, relative rather than absolute frequency.

Mass-participation events, varying from music festivals to ultramarathons, are now well-established features in the spectrum of recreation in the countryside. The structured nature of these events probably results in most participants interacting with the landscape they occupy in a predictable (in spatial and temporal terms at least) manner. Thus, from the perspective of infection epidemiology, mass-participation events provide opportunities to easily collect large numbers of samples (which would otherwise require considerable effort to accumulate) over a short period. In this study, we demonstrate the potential of this approach by using a mass-participation event to quantify the frequency with which those engaged in outdoor pursuits in tick-infested areas actually get bitten.

## Materials and Methods

### Sample Collection 

The Lowe Alpine Mountain Marathon (LAMM) is an annual long-distance mountain running event held in the Scottish Highlands. Teams of 2 runners each navigate mountainous terrain, carrying all their equipment for an overnight camp. To suit competitors with different levels of expertise and fitness, the event comprises 2 days of racing over several routes of varying lengths, typically 20–40 km. In June 2014, the LAMM took place around Wester Ross on the west coast of mainland Scotland (http://www.lamm.co.uk/2014/LAMM2014Map.jpg); the competition area covered ≈500 km^2^. Racing on the first day was completed by 624 competitors, of whom 566 went on to complete racing on the second day. Before the event, the objectives and methods of our study were circulated to all competitors via a LAMM website (no longer available) and were reiterated to competitors in person the night before the race. As competitors passed through the finish area on the first day, they were given a 1.5-mL tube, containing 70% ethanol and labeled “1” and were asked to place any ticks they found on their bodies into the tube and then put the tube into a bin provided at the overnight midway camp. We also offered a tick-removal service, which proved popular and served to encourage wider participation. As competitors passed through the finish area on the second day, they were given an addressed envelope containing a 1.5-mL tube containing 70% ethanol and labeled “2.” Competitors were asked to check themselves either immediately or when they returned home, to place any ticks found in the tube as before, and then to put the envelope containing the tube in the mail. After we processed the samples and collated the data, we posted a summary of survey results (at http://www.lamm.co.uk/2014/LAMM_LymeResults_2014.pdf), and the link was emailed to all competitors. This email also asked any competitor in whom Lyme borreliosis developed after the LAMM to contact the study team.

To assess if borreliae were an environmental hazard in the area and provide an indication of the scale of this hazard, we surveyed the competition area of the event for questing ticks by blanket dragging at 3 sites, specifically the day-1 assembly area (rough pasture used for sheep grazing) surveyed the night before the event, the overnight midway camp (heather, long grass), and pine woodland in Lochcarron, located ≈2 km from the assembly area (both surveyed during event) (Table). These efforts also served to increase awareness of the ongoing study throughout the event. Each site was surveyed for 30 minutes by repeatedly dragging a 1-m^2^ wool blanket over the vegetation. After each drag, all ticks were removed from the blanket and placed in 70% ethanol.

**Table:** Numbers of ticks collected and prevalence of *Borrelia burgdorferi* infections among those ticks at 3 Lowe Alpine Mountain Marathon sites, Scotland, 2014

Location	No. ticks collected	No. (%) nymphs *containing B. burgdorferi* DNA
Lochcarron	2 larvae,156 nymphs, 6 adults	11 (7.1)
Day-1 assembly area	0 larvae, 107 nymphs, 0 adults	16 (14.9)
Midway camp	0 larvae, 20 nymphs, 2 adults	2 (10.0)

### Identification of Ticks and Determination of Infection Status

We recorded the life stages of all ticks and examined them microscopically to identify their species. A DNA extract was prepared from each tick and then incorporated as template into a real-time PCR, originally described for the detection of *B. burgdorferi* s.l. but subsequently found to have a specificity that includes *B. miyamotoi* (*8*). To delineate *Borrelia* species/genospecies, we incorporated as template extracts that yielded a product in this reaction into a *B. burgdorferi* s.l.–specific nested PCR (*9*) and a *B. miyamotoi–*specific real-time PCR (*10*). Products of the first of these reactions were sequenced, and unambiguous sequence data were used to determine *Borrelia* genospecies (*9*). Extraction/cross-contamination controls were co-processed with ticks at a ratio of 1:4. Each PCR incorporated positive and negative (reagents only) controls. PCRs were prepared in a dedicated laboratory in which PCR products were never handled.

## Results

### Survey 

The species of all ticks encountered in our study was *I. ricinus*. On day 1 of the LAMM, 217 ticks were removed and submitted by 53 competitors, and on day 2 (or later by mail), 347 ticks were removed and submitted by 78 competitors. It is not known how many competitors submitted ticks on both days, but on the basis of a recorded 624 competitors on day 1 and 566 on day 2, we an estimate that a minimum of ≈8.5% of competitors on day 1 and 13.8% of competitors on day 2 were bitten by ticks. Significantly more competitors were bitten on day 2 than on day 1 (χ^2^ 8.47, 1 df, p<0.01). We collected 98 submissions containing 153 nymphs. A total of 44 (7.1%) competitors on day 1 and 54 (9.6%) competitors on day 2 were bitten by nymphs. We collected 75 submissions containing 411 larvae. A total of 25 (4.0%) competitors on day 1 and 50 (8.8%) competitors on day 2 were bitten by larvae. Both nymphs and larvae were removed from the skin of 16 (2.6%) competitors on day 1 and 26 (4.6%) competitors on day 2. Each submission contained 0–28 larvae and 0–9 nymphs, although most submissions contained either only 1 nymph or 1 larva.

### *Borrelia* Infection Prevalence and Diversity among Ticks 

Infection with *B. burgdorferi* s.l. was found in 3 (0.7%) of larvae and 19 (12.4%) of 153 nymphs that had fed on competitors. We were able to further characterize 16 of these ticks: 1 larva was infected with *B. afzelii*, 1 larva and 2 nymphs with *B. burgdorferi* sensu stricto, 7 nymphs with *B. valaisiana,* 2 nymphs with *B. garinii*, 1 nymph with *B. miyamotoi*; and 2 nymphs had mixed infections (1 with *B. valaisiana* and *B. garinii* and the other with *B. afzelii* and *B. garinii*). Infected larvae were removed from 2 competitors, and infected nymphs were removed from 16 competitors. In 4 instances, >1 tick removed from the same competitor was infected. A total of 10 infected ticks (3 larvae and 7 nymphs) were removed from 9 (1.4%) of the 624 day-1 competitors, whereas 12 infected nymphs were removed from 9 (1.6%) of the 566 day-2 competitors. No competitor reported development of Lyme borreliosis or symptoms compatible with *B. miyamotoi* infection subsequent to the LAMM.

### Survey of the Environment

A total of 283 questing nymphal ticks were collected from the competition area and tested for the presence of DNA belonging to *Borrelia* spp. The overall prevalence of *B. burgdorferi* s.l. infection was 10.2% (29/283) (Table). We were able to further characterize infections in 11 of these ticks, finding 7 to be infected with *B. afzelii*, 3 with *B. garinii*, and 1 with *B. valaisiana*. The prevalence of infection among nymphs removed from humans did not differ significantly from that among questing ticks (χ^2^ 0.75, 1 df, p = 0.39).

## Discussion

The presence of ticks at the site of the 2014 LAMM was entirely expected, as was presence of *B. burgdorferi* s.l. infections in these ticks. In a recent survey of 25 sites across Scotland, primarily in the Highlands, infected ticks were found at all sites (*11*). The overall prevalence of *B. burgdorferi* s.l. infections in questing nymphs and in nymphs removed from competitors (48/436, 11.0%) and the diversity of *B. burgdorferi* s.l. genospecies encountered was akin to that previously reported in Scotland and elsewhere in the United Kingdom (*8*,*11*). The presence of *B. miyamotoi* in Scotland has not been reported previously, but a study of questing nymphs in England suggested that the pathogen is widespread, albeit at a very low prevalence (3/954, 0.3%) (*12*).

Perhaps the most unexpected observation was the high proportion of competitors bitten by ticks; our estimates are that 8.5% were bitten on day 1 and 13.8% on day 2. Of 131 competitors who reported being bitten by ticks, 33 (≈25%) found larvae only. This life stage is often overlooked with regard to *B. burgdorferi* s.l. transmission, but our detection of *B. burgdorferi* s.l. DNA in 0.7% of larvae from competitors supports the work of others (*13*–*15*), indicating that although rates of infection may be relatively low among larvae, ticks at this life stage should not be disregarded as a source of Lyme borreliosis. Low prevalence of *B. miyamotoi* infections in *I. ricinus* larvae collected in mainland Europe has been demonstrated (*15*). Our failure to detect this species in any of the 411 larvae we tested indicates that, on the event site at least, infection of larvae is extremely rare. The remaining 98 submissions, representing 75% of all those bitten by ticks, found either nymphs only or both nymphs and larvae. Nymphs have far greater potential to transmit *B. burgdorferi* s.l.; in this study, prevalence of infection among nymphs was ≈18 times higher than that among larvae. Of 98 nymph submissions, 16 (16%) were infected with either *B. burgdorferi* s.l. or *B. miyamotoi*; 1.1% of day-1 competitors and 1.6% of day-2 competitors removed infected ticks from their bodies.

Over the 2 days of the LAMM, competitors assembled at the camping area at 6 pm on the evening before the start and departed after finishing the race 2 days later. Given that we encountered ticks not just on the LAMM course but also in the camping area used by the competitors when not racing, we can reasonably assume that the only periods when competitors were not exposed to ticks was when they were inside their tents. We therefore estimate that through the 48 hours of the event, 624 competitors were exposed to ticks for ≈32 hours, a total of ≈20,000 competitor-hours. The total of 564 tick bites occurring during this period indicate 1 bite every 35 competitor-hours. Of these, 153 were from nymphs; thus, 1 nymph tick bite occurred every 131 competitor-hours; a bite from an infected nymph occurred once every 1,051 competitor-hours. 

These data provide an early quantification of the frequency of human exposure to *B. burgdorferi* s.l. in the vicinity of the LAMM and, given the estimated abundance of nymphal ticks in this area and the prevalence of *B. burgdorferi* s.l. infections among these ticks, may represent values encountered more widely across Scotland and other parts of the United Kingdom (*8*,*11*). Extrapolation of these data may offer a tentative insight into frequency of human exposure in similar tick-infested habitat across the country. However, as well as caveats associated with geographic/spatial variation in the environmental hazard posed by *B. burgdorferi* s.l., other determinants of tick exposure need also to be considered, including anthropogenic determinants. For example, the degree to which competitors’ behavior was representative of countryside users at large (or a specific cohort thereof) is unknown; the infrequency with which LAMM competitors used clear footpaths may have resulted in their encountering questing ticks more often, and their light apparel (many competitors wore shorts and short-sleeved tops) may have enabled attachment of more ticks. In addition, abiotic factors such as the influence of local weather conditions on tick questing behavior during the LAMM and the topography of the course, sections of which were at relatively high altitudes where ticks are less abundant (*16*), need to be considered. Of note, we observed more frequent tick bites on the second day of competition, despite persistent light rain throughout the morning, an observation in keeping with previous reports that ticks remain active in rain and that rainfall is associated with increased tick activity (*17*). However, we cannot rule out that this observation resulted from competitors simply being more vigilant after the competition finished.

Various approaches to exploring the frequency of human exposure to ticks have previously been taken. These approaches vary from the quantification of ticks accumulated on fabricated leggings worn by investigators moving over/through tick-infested vegetation (thus, rate of encounter rather than attachment) (*1**,**18*) to assessment of the number of patients seeking help from medical staff for tick removal; in 1 such survey of visitors to a popular woodland site in southern England during April–October 1996 and 1997, ticks were removed from almost 1,100 persons (*19*). However, only very few studies have attempted to quantify the rate at which users of tick-infested habitat actually get bitten. One survey, of 568 soldiers at an outdoor training base in Germany, recorded 710 tick bites during April–September 2009, a mean incidence rate of 2.3 bites/1,000 person-days (*20*). More recently, a survey of 931 scouts attending summer camps in Belgium yielded a mean incidence rate of 22.8 tick bites/1,000 person-days (*21*). Both of these incidence rates are markedly lower than that reported in the current study, which equates to 677 bites/1,000 competitor-days. Why such variation exists is unclear; it may be artifactual, resulting from differences in the efficiency of tick detection in 3 studies, or it may be genuine. If the latter, it is worth considering that our survey was conducted in early June, when the abundance of immature ticks is probably near its seasonal peak (*22*), whereas the surveys in Germany and Belgium extended over several months, during some of which tick abundance is probably reduced. Indeed, in the study in Germany, >95% of all tick bites occurred during 3 of the 6 months (April–September) surveyed (*20*). Also, in Belgium, marked differences in incidence rates were observed between camps; a range of 0–97.9 bites/1,000 person-days were recorded (*21*). None of the 3 surveys under discussion included accurate estimates of questing tick densities at the survey sites; however, given that the questing tick densities at the 3 LAMM sites surveyed were not unusually high (mean of 189 ticks/person-hour, which equates to 98/100 m^2^ (*23*), it is unlikely that the ≈100-fold variation in mean incidence rate of tick bites between our study and that of Faulde et al. (*20*) is solely a reflection of variation in local tick abundance. Perhaps more pertinent is that the participants of the study in Germany wore battle dress uniforms and were provided with arthropod-repellent skin cream, whereas the LAMM competitors wore light apparel, frequently shorts and short-sleeved tops, thus making it easier for ticks to access their skin (and possibly attracting ticks more by unimpeded release of attractant molecules from the skin). De Keukeliere et al. (*21*) did not provide details of what the participants in their study wore or the nature of their activities, but given that they were all scouts 8–16 years of age, it is reasonable to assume their clothing was more akin to that of LAMM competitors than that of German soldiers.

That none of the competitors reported clinical symptoms compatible with either *B. burgdorferi* s.l. or *B. miyamotoi* infections subsequent to the LAMM is most likely the result of the well-established prophylactic benefits of removing ticks within a few hours of attachment. However, it may also reflect a low transmission rate of *B. burgdorferi* s.l. from ticks to humans (*19*,*24*) or a low incidence of clinical manifestations among those infected (*25*,*26*).

In summary, our study demonstrates how mass-participation outdoor recreational events can be used to assess human exposure to dangers such as tickborne pathogens. By using one such event, we were able to survey a large number of persons very efficiently; we obtained more samples in just 2 days than previous studies with similar aims (*19*,*20*) obtained in 6 months. Mass-participation outdoor recreational events are now more diverse and popular than ever; for example, thousands of orienteers and mountain/hill/trail runners participate in events every weekend, and vast numbers attend music or arts festivals throughout the summer. Furthermore, participants generally have a strong connection with the outdoors and so are probably willing contributors to scientific studies linked to nature. A refinement to our approach would be to track more precisely the movement of persons through the landscape. This tracking could be achieved by focusing on events at which participants follow a fixed course or wear GPS (global positioning system) tracking devices (as is becoming increasingly common), akin to a study quantifying the risk for tick infestation of dogs (*27*).
